# Antidepressant Use in Depressed Women During Pregnancy and the Risk of Preterm Birth: A Systematic Review and Meta-Analysis of 23 Cohort Studies

**DOI:** 10.3389/fphar.2020.00659

**Published:** 2020-05-19

**Authors:** Qing Chang, Xiao-Yu Ma, Xin-Rui Xu, Han Su, Qi-Jun Wu, Yu-Hong Zhao

**Affiliations:** ^1^Department of Clinical Epidemiology, Shengjing Hospital of China Medical University, Shenyang, China; ^2^Clinical Research Center, Shengjing Hospital of China Medical University, Shenyang, China

**Keywords:** antidepressants, depression, drug safety, meta-analysis, preterm birth

## Abstract

**Objective:**

The associations between maternal use of antidepressant during pregnancy and preterm birth (PTB) has been the subject of much discussion and controversy. The aim of the present study was to systematically review the association between antidepressant use during pregnancy and the risk of PTB, especially in depressed women.

**Methods:**

A computerized search was conducted in PubMed, PsycINFO, and Embase before *June 30, 2019*, supplemented with a manual search of the reference lists, to identify original research regarding PTB rates in women taking antidepressants during pregnancy. A random-effects model was used to calculate the summarized relative risks (RRs) and 95% confidence intervals (CIs). The potential for publication bias was examined through Begg' s and Egger' s tests.

**Results:**

A total of 2,279 articles were reviewed, 23 of which were selected. The risk of PTB was increased in women with depression [1.58 (1.23−2.04)] and in the general pregnant female population [1.35 (1.11−1.63)] who used antidepressants during pregnancy. Similar results were observed in depressed women treated with selective serotonin reuptake inhibitors (SSRIs) during pregnancy [1.46 (1.32−1.61)]. There was no significantly increased risk of PTB observed with SSRI use in the general pregnant female population [1.25 (1.00−1.57)], and the heterogeneity of these studies was high.

**Conclusions:**

The results of this meta-analysis indicate maternal antidepressant use is associated with a significantly increased risk of PTB in infants. Health care providers and pregnant women must weigh the risk-benefit potential of these drugs when making decisions about whether to treat with antidepressant during pregnancy.

## Introduction

Preterm birth (PTB), defined as delivery prior to 37 weeks of gestation, is regarded as a global public health concern, since it is the leading cause of infant mortality worldwide (Blencowe et al., 2012). In comparison with infants born full-term, PTB infants are at a greater risk of multiple health problems, including neurological and long-term developmental disorders (Marlow et al., 2000). Rates of PTB have fortunately declined over the last decade, occurring in approximately 12% of pregnancies in the U.S. (Purisch and Gyamfi-Bannerman, 2017). This decline is due, in part, to declines in the number of births to teens and young mothers. The annual economic cost related to PTB has been approximated at $26 billion dollars in the U.S. and £939 million in the U.K. (Mangham et al., 2009).

Many factors are thought to contribute to the increased PTB rate, including a higher mean maternal age, more frequent use of assisted reproductive technologies and a resulting increase in multiple gestations, and higher rates of preterm inductions and cesarean deliveries (Chang et al., 2013). Interestingly, since the prevalence of depression during pregnancy has increased from 8.3% to 12.7%, many studies have shown that antidepressant use during pregnancy increases the risk of PTB (Lee et al., 2007; El Marroun et al., 2014; Staneva et al., 2015). Additionally, there exists emerging evidence that maternal depression during pregnancy is a risk factor for PTB (El Marroun et al., 2014; Staneva et al., 2015). However, based on the existing literature, it is difficult to draw a unified conclusion regarding the association of antidepressant use during pregnancy and PTB, since the research differs in terms of the timing of antidepressant use during pregnancy, adjustment for potential confounding variables, inclusion of lifestyle factors, presence of comorbidities, and severity of the underlying psychiatric illness. The existing four published meta-analyses (Huybrechts et al., 2014; Ross and Grigoriadis, 2014; Hsiang and Bridge, 2014; Eke et al., 2016) had several limitations. Firstly, these meta-analyses consisted of both retrospective and prospective cohort studies, the former of which may have caused recall bias. Moreover, in these meta-analyses, there were no reports of depressed pregnant women in the study population (Huybrechts et al., 2014; Ross and Grigoriadis, 2014; Hsiang and Bridge, 2014), with the exception of one study (Eke et al., 2016) including eight original studies that examined the effect of selective serotonin reuptake inhibitor (SSRI) use on PTB in pregnant women with depression. Additionally, information regarding subgroup analyses stratified by geographic locations and whether adjustments were made for confounders [e.g., maternal age, ethnicity, education, smoking, alcohol consumption, body mass index (BMI), and parity] were not provided.

Therefore, in the present study, a meta-analysis of all available studies was conducted to quantitate the strength of the relationship between antidepressant use during pregnancy and PTB especially in depressed women.

## Materials and Methods

### Search Strategy

The PubMed, PsycINFO, and Embase databases were searched for related articles published before *June 30, 2019*. The search queries used are listed in **Supplementary File 1**. The articles were imported into the NoteExpress library for removal of the duplicates.

### Study Selection

Eligibility criteria were defined as: (1) articles available in English with the full text provided; (2) studies conducted in humans; (3) prospective cohort studies; (4) antidepressant use as the study exposure and PTB as the study outcome; (5) antidepressant use during pregnancy; (6) no psychotropic drug use in the control group; and (7) reported risk ratio or adequate data for the calculation of an effect size as a relative risk (RR) between antidepressant use and PTB.

### Data Extraction

The study quality of eligible articles was assessed by two reviewers (X-YM and X-RX), who subsequently extracted data from each selected article including the study design, sample size, data source, criteria for inclusion/exclusion, definition of exposure, definition of outcome, outcomes with risk estimates and 95% confidence intervals (CIs), and adjusted confounders. The two reviewers extracted data independently and any disagreements were resolved by discussion with a third reviewer (QC), when necessary.

For one study (Källén, 2004) that characterized neonates in the Swedish birth registry following maternal antidepressant use in late pregnancy, discussion among the three reviewers resulted in recalculation of the crude RR value according to the control group as the non-exposed population.

### Risk of Bias Assessment

The two reviewers independently performed a quality assessment using the Newcastle−Ottawa scale (NOS) (Wells et al., 2011) for cohort studies. The scale of this quality tool consists of three components for which a maximum of 9 points can be given: selection of study participant groups (max. 4 points); comparability of study groups (max. 2 points); and ascertainment of outcome (max. 3 points). Studies were considered to have a low risk of bias if they achieved a full rating in at least two of these categories (Odutayo et al., 2016).

### Statistical Analysis

STATA, version 11.0 (StataCorp, College Station, TX) was used for all statistical analyses. The mean effect size approach was used to pool estimates, which has been applied in other studies (McDonald et al., 2010; Gao et al., 2018). The effect size was weighted as per the study sample size. Estimates were pooled using the DerSimonian and Laird random-effects model to calculate the summarized RRs and 95% CI, as large inter-study heterogeneity was expected (DerSimonian and Laird, 1986). For studies reporting the necessary data instead of providing the risk estimates directly, these data were used to calculate the crude RRs. The *I*^2^ value represents the percentage of total variation across studies due to heterogeneity rather than chance (Higgins and Green, 2011). Values of 25%, 50%, and 75% are regarded as representing low, moderate, and high heterogeneity (Higgins et al., 2003). A two-tailed *P*-value <0.05 was considered statistically significant. Potential publication bias was assessed using Begg's test (Begg and Mazumdar, 1994).

If more than eight studies were available, potential sources of heterogeneity were explored by conducting subgroup analyses according to the following parameters: geographic location (Europe vs. North America or other regions) and adjustment for potential confounders (adjusted vs. unadjusted) including maternal age, ethnicity, maternal education, tobacco use, alcohol consumption, BMI during pregnancy, and parity. Heterogeneity between subgroups was evaluated by meta-regression analysis. To determine the influence of individual studies on each analysis of the estimated RR, sensitivity analysis was conducted that recalculated the pooled effect by omitting one study at a time. The population was divided into four groups: antidepressant use in the general pregnant female population, antidepressant use in pregnant women with depression, SSRI use in the general pregnant female population, and SSRI use in pregnant women with depression.

## Results

### Search Results

**Figure 1:**
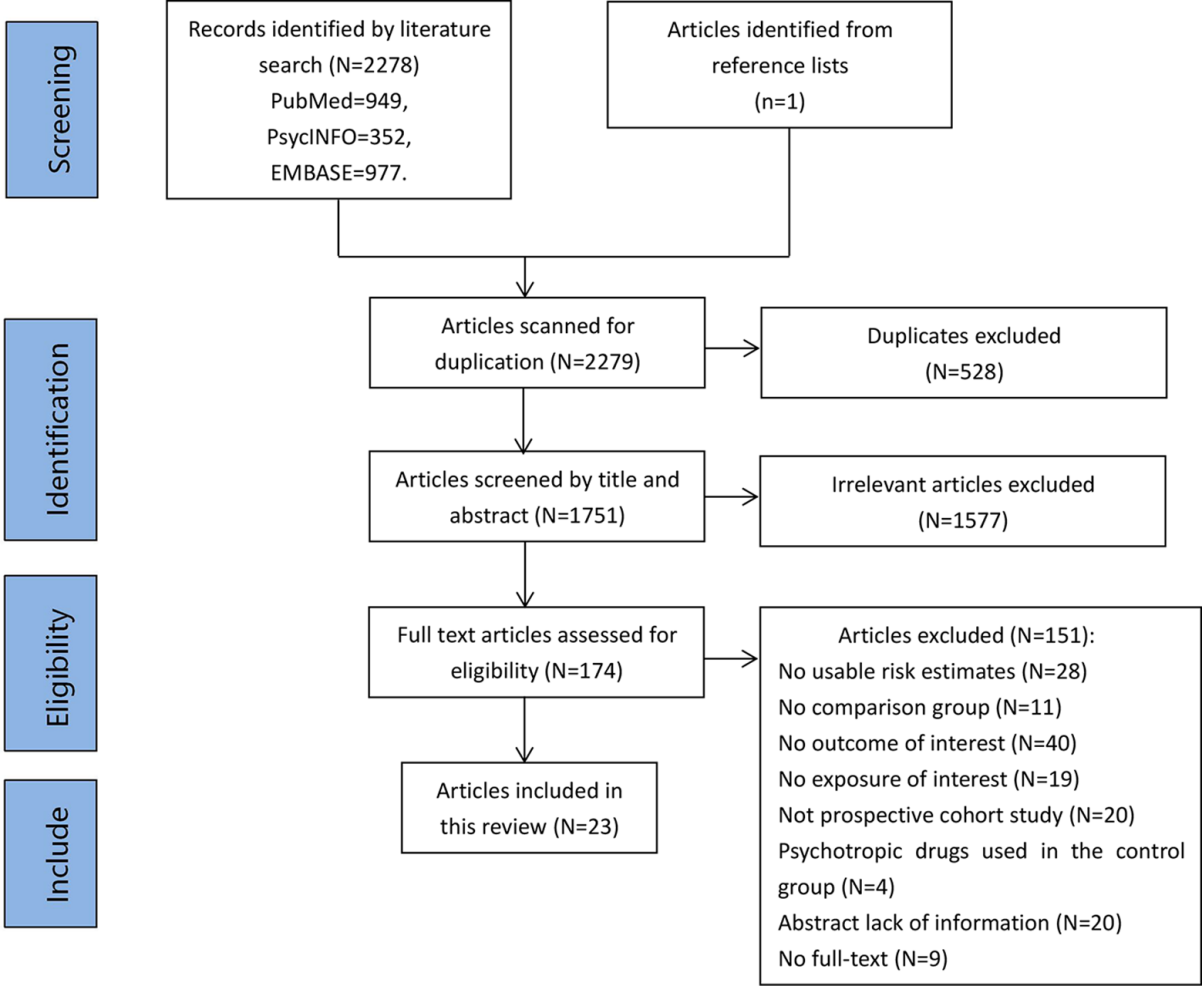
Flowchart of the article selection process.

A total of 2,278 potentially eligible articles were identified in PubMed, PsycINFO, and Embase. One additional study was added following a manual search of the reference lists. Following removal of 528 duplicate articles, the titles and abstracts were screened, and 1,577 irrelevant articles were removed. A total of 174 full-text articles were assessed for eligibility, and 23 (published between 2004 and 2019) were ultimately included in the present analysis. A flow diagram of study identification is shown in **Figure 1**.

All prospective cohort studies assessing antidepressant exposure during pregnancy were included. Eight studies (Calderon-Margalit et al., 2009; Lund et al., 2009; El Marroun et al., 2012; Nordeng et al., 2012; Viktorin et al., 2016; Laine et al., 2019; Richardson et al., 2019) were conducted in Europe, 13 (Källén, 2004; Sivojelezova et al., 2005; Djulus et al., 2006; Suri et al., 2007; Wisner et al., 2009; Einarson et al., 2010; Einarson et al., 2011; Latendresse and Ruiz, 2011; Yonkers et al., 2012; Klieger-Grossmann et al., 2012; Sadowski et al., 2013; Winterfeld et al., 2015; Yonkers et al., 2017) in North America, 1 in Asia and southeast Europe (Şahingöz et al., 2014), and 1 in Australia (Lewis et al., 2010). The included studies all defined PTB as gestational weeks <37, with the exception of one study, Malm H, 2015 (Malm et al., 2015), which classified PTB into two types [late preterm (32−36 gestational weeks) and very preterm (< 32 weeks) births]. We did the analysis after combining them. Most studies evaluated the association between SSRI use and the risk of PTB (n = 12) (Källén, 2004; Sivojelezova et al., 2005; Suri et al., 2007; Wisner et al., 2009; McDonald et al., 2010; El Marroun et al., 2012; Yonkers et al., 2012; Huybrechts et al., 2014; Şahingöz et al., 2014; Malm et al., 2015; Viktorin et al., 2016; Yonkers et al., 2017); four studies evaluated the association between the use of all antidepressants and PTB (Suri et al., 2007; Einarson et al., 2010; Nordeng et al., 2012; Laine et al., 2019); four studies evaluated the association between tricyclic antidepressant (TCA) (Källén, 2004), mirtazapine (Djulus et al., 2006; Winterfeld et al., 2015), and venlafaxine (Richardson et al., 2019) use and PTB; three studies evaluated the association between the use of one or more antidepressants (Calderon-Margalit et al., 2009; Lewis et al., 2010; Einarson et al., 2011) and PTB; and one study evaluated the association between second-generation antipsychotics (SGAs) (Sadowski et al., 2013) (e.g., SSRIs, benzodiazepines, anticonvulsants, SNRIs, atypical antidepressants) and PTB. Eight studies recruited patients through the Teratology Information Service (Sivojelezova et al., 2005; Djulus et al., 2006; Einarson et al., 2010; Einarson et al., 2011; Klieger-Grossmann et al., 2012; Sadowski et al., 2013; Winterfeld et al., 2015; Richardson et al., 2019) and four studies recruited participants from the national birth registry (Källén, 2004; Viktorin et al., 2016; Laine et al., 2019). The characteristics of the included studies are shown in **Table 1**.

**Table 1:** Characteristics of the studies included in the meta-analysis.

Author	Country	Year	Study type	Data period	Definition of preterm	Exposure	Sample size (preterm)	Adjusted confounders
Richardson et al., 2019	UK	2019	UKTIS	1995−2018	—	Venlafaxine	1,274 (158)	Venlafaxine-exposed pregnancies by both calendar year and maternal age (each ±2 years) at UKTIS referral
Laine et al., 2019	Finnish	2019	National birth registry	2009−2015	<37	All ADs	6263	Age, cohabitation, smoking, education, body mass index, fertility treatments, previous pregnancies, and gestational diabetes mellitus
Yonkers et al., 2017	Connecticut and southern Massachusetts	2017	U.S. prospective cohort	2005−2009	<37	SRIs	2,654 (225)	Age, race/ethnicity, educational level, and smoking, heavy drinking, and illicit drug use during pregnancy
Viktorin et al., 2016	Swedish	2016	National birth registry	2006−2009	<259 days	SSRIs	390,404	Mother's education, mother's BMI, parity, mother's age at pregnancy, mother's previous psychiatric history, mother's smoking status at the first visit to maternal care
Winterfeld et al., 2015	Canada	2015	TIS	1995−2011	<37	Mirtazapine	581 (57)	Selected randomly, and cases and control subjects were matched by TIS center, year of TIS contact (± 2 years), maternal age (± 2 years), and gestational age at the time of call (± 4 weeks)
Malm et al., 2015	Finland	2015	National birth registry	1996−2010	Late preterm (32−36 weeks)	SSRIs	56,775 (2,449)	Gender and birth period (1996–2000, 2001–2005, and 2006–2010), maternal age at delivery, place of residence, marital status, parity, smoking, socioeconomic status, purchase of anxiolytics, sedative-hypnotics, or antiepileptic drugs, pre-pregnancy diabetes, and other chronic diseases
					Very preterm (< 32 weeks)		56,775 (359)	
Şahingöz et al., 2014	Konya and Istanbul	2014	Turkish prospective cohort	—	20 < weeks < 37	SSRIs	89	—
Sadowski et al., 2013	Canada	2013	TIS	2005−2009	<37	Second-generation antipsychotics	266 (17)	Age at conception (± 3 years) and pregnancy duration at the initial time of contact (± 2 weeks)
Nordeng et al., 2012	Norwegian	2012	The MoBa study	2000−2006	<37	All ADs	62,347	Level of depression, maternal age at delivery, education, parity, pre-pregnancy BMI, maternal asthma or cardiovascular disease, NSAID use, folic acid use, and smoking during pregnancy
Yonkers et al., 2012	Connecticut and southern Massachusetts	2012	U.S. prospective cohort	2005−2009	<37	SRIs	2,654 (225)	Age, education, race, smoking, illicit drug use and pregnancy history, number of lifetime hospitalizations, age of depressive onset, number of prior depressive episodes, post-traumatic stress disorder, generalized anxiety disorder, panic disorder in pregnancy, and suicidal thoughts in pregnancy
El Marroun et al., 2012	Netherlands	2012	The Generation R study	2002−2006	<37	SSRIs	7,126 (365)	Maternal age at intake, gender of the child, maternal education, ethnicity, maternal smoking and drinking habits, body mass index, parity, and maternal benzodiazepine use
Klieger-Grossmann et al., 2012	Canada	2012	TIS	—	<37	Escitalopram	425 (28)	Maternal age ±2 years, alcohol consumption and smoking, and gestational age at the time of call ±2 weeks
Einarson et al., 2011	Canada	2011	TIS	1992−2007	<37	All ADs	178 (15)	Maternal age (± 2 years), smoking and alcohol use, time of call to Motherisk
Latendresse and Ruiz, 2011	Utah	2011	U.S. prospective cohort	2007.3−11	<37	SSRIs	100	—
Einarson et al., 2010	Canada	2010	TIS	—	< 37	All ADs	1,856 (132)	Maternal age (± 2 years), alcohol, tobacco, concurrent drug use
Lewis et al., 2010	Australia	2010	Clinic-based prospective cohort	2004−2005	<37	SSRIs/SNRIs/NaSSAs	54 (5)	—
Lund et al., 2009	Denmark	2009	Aarhus birth prospective cohort	1989−2006	<37	SSRIs	57,001 (2796)	Maternal age, body mass index, smoking, a previous pregnancy with prematurity, and parity
Wisner et al., 2009	Cleveland and Pitts burgh	2009	U.S. prospective cohort	2000−2001, 2003−2007	Late preterm (≥34 to <37 weeks)	SSRIs	279 (24)	Maternal age and race
					Early preterm (< 34 weeks)			
Calderon-Margalit et al., 2009	Swedish	2009	Omega study	since 1996	20 < weeks < 37	SSRIs/SSRI+SNRI	2,631 (253)	Maternal age, race, years of education, marital status, smoking during pregnancy, preeclampsia, parity, and singleton/multiple pregnancy
Suri et al., 2007	Los Angeles	2007	the University of California	2000−2005	—	All ADs	90 (14)	—
Djulus et al., 2006	Canada	2006	TIS	2002−2005	<37	Mirtazapine	208 (12)	Maternal age at the time of conception (± 2 years), gestational age at the first contact (± 2 weeks), tobacco use, alcohol consumption, and chronic conditions
Sivojelezova et al., 2005	Canada	2005	TIS	1999-2002	<37	Citalopram	264 (16)	Maternal age (± 2 years), gestational stage of pregnancy (± 2 weeks) at the time of recruitment
Källén, 2004	Swedish	2004	National birth registry	1995−2001	<37	Tricyclic	563,656 (28,743)	Year of birth, maternal age, parity, and maternal smoking in early pregnancy
						SSRIs		

TIS, Teratogen Information Service; AD, antidepressant; SSRI, selective serotonin reuptake inhibitor; SRI, serotonin reuptake inhibitor; NaSSA, noradrenergic and specific serotonergic antidepressant; NSAID, nonsteroidal anti-inflammatory drug; SNRI, serotonin–norepinephrine reuptake inhibitor; UKTIS, UK Teratology Information Service.

### Bias Assessment

Analysis of the included studies using Newcastle−Ottawa criteria indicated that 21 studies were at a low risk and two studies (Källén, 2004; Latendresse and Ruiz, 2011) were at a high risk of bias. All studies achieved a total score of 6−9 (median = 7) (**Supplementary File 2**).

### Antidepressant Use in the General Pregnant Female Population

**Figure 2:**
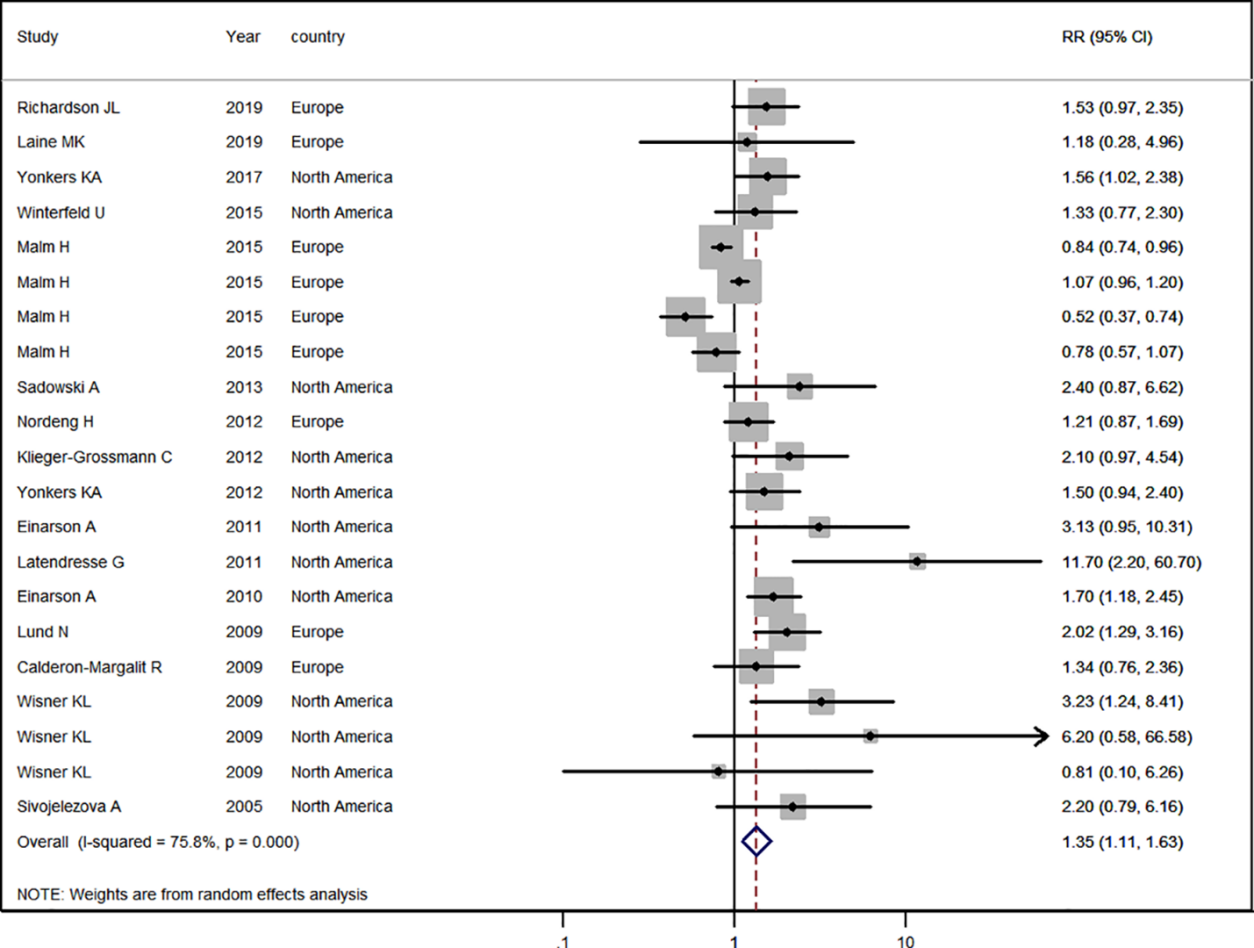
Meta-analysis of antidepressant use in pregnant women and the risk of preterm birth.

Sixteen studies (Källén, 2004; Sivojelezova et al., 2005; Lund et al., 2009; Calderon-Margalit et al., 2009; Wisner et al., 2009; Einarson et al., 2010; Einarson et al., 2011; Latendresse and Ruiz, 2011; Nordeng et al., 2012; Klieger-Grossmann et al., 2012; Sadowski et al., 2013; Winterfeld et al., 2015; Malm et al., 2015; Yonkers et al., 2017; Richardson et al., 2019; Laine et al., 2019) in the general pregnant female population were included for this analysis. The adjusted RR was 1.35 (95% CI 1.11−1.63, *I*^2^ = 75.8%, *P* < 0.001, **Figure 2**). A significantly increased risk of PTB was observed with antidepressant use in the general pregnant female population. Subgroup analysis of antidepressant use and the risk of PTB is shown in **Table 2**. In 10 North American studies, the adjusted RR was 1.78 (1.45−2.18). Following adjustment for maternal age, the RR was statistically significant [1.28 (1.06−1.54)]. If tobacco use and parity were not adjusted for, the RRs were statistically significant [2.01 (1.35−2.98), 1.75 (1.45−2.11)]. There was no evidence of publication bias in any of the studies.

**Table 2:** Subgroup analysis of antidepressant use in the general pregnant women and the risk of preterm birth: results of the meta-analysis.

	**Antidepressant**				
	**No. of studies**	**Summary RR (95% CI)**	***I*^2^, %**	P*	P**
Geographic location					0.01
Europe	6	1.04 (0.84–1.23)	79.7	< 0.001	
Northern America	10	1.78 (1.45–2.18)	5.3	0.39	
Adjustment for confounders					
Maternal age					0.05
Yes	14	1.28 (1.06–1.54)	74.9	< 0.001	
No	2	4.60 (0.99–21.19)	60.8	0.11	
Ethnicity					0.39
Yes	4	1.59 (1.22–2.06)	0.0	0.52	
No	12	1.27 (1.02–1.57)	79.5	< 0.001	
Maternal education					0.92
Yes	5	1.36 (1.11–1.68)	0.0	0.90	
No	11	1.36 (1.07–1.72)	80.0	< 0.001	
Tobacco use					0.05
Yes	6	1.19 (0.97–1.45)	79.4	< 0.001	
No	10	2.01 (1.35–2.98)	30.7	0.18	
Alcohol consumption					0.22
Yes	4	1.73 (1.34–2.24)	0.0	0.70	
No	12	1.24 (1.01–1.52)	75.5	< 0.001	
Pregnancy BMI					0.55
Yes	2	1.93 (1.26–2.95)	0.0	0.48	
No	14	1.31 (1.07–1.59)	75.7	< 0.001	
Parity					0.01
Yes	5	1.03 (0.83–1.28)	81.8	< 0.001	
No	11	1.75 (1.45–2.11)	0.0	0.46	

BMI, body mass index; CI, confidence interval; RR, relative risk.

*P for heterogeneity within each subgroup.

**P for heterogeneity between subgroups with meta-regression analysis.

### Antidepressant Use in Pregnant Women With Depression

**Figure 3:**
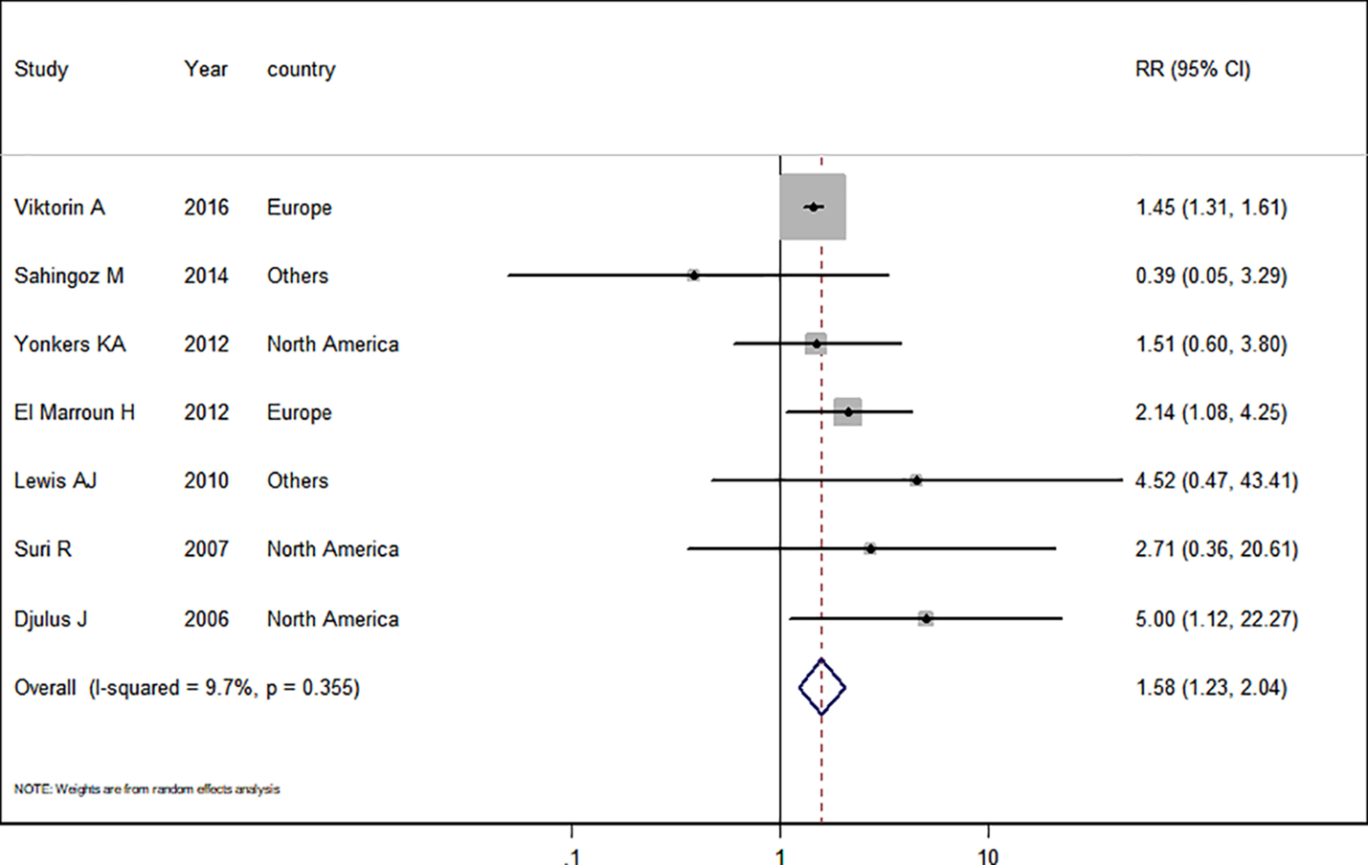
Meta-analysis of antidepressant use in pregnant women with depression and the risk of preterm birth.

Seven studies (Djulus et al., 2006; Suri et al., 2007; Lewis et al., 2010; Yonkers et al., 2012; El Marroun et al., 2012; Şahingöz et al., 2014; Viktorin et al., 2016) in the pregnant women with depression were included for this analysis. The adjusted RR was 1.58 (95% CI 1.23−2.04, *I*^2^ = 9.7%, *P* = 0.365, **Figure 3**). A significantly increased risk of PTB was observed when the analysis was restricted to pregnant women with depression. Subgroup analysis of antidepressant use in pregnant women with depression and the risk of PTB was shown in **Table 3**. In two European studies, the adjusted RR was 1.51 (95% CI 1.20−1.91). Following adjustment for maternal age, ethnicity, maternal education, tobacco use, BMI during pregnancy, and parity, the adjusted RRs were statistically significant: 1.62 (1.20−2.18), 1.89 (1.09−3.28), 1.46 (1.32−1.62), 1.62 (1.20−2.18), 1.51 (1.20−1.91), and 1.46 (1.32−1.62), respectively. There was no evidence of publication bias in any of the studies.

**Table 3:** Subgroup analysis of antidepressant use in pregnant women with depression and risk of preterm birth: results of meta-analyses.

	Antidepressant use				
	No. of studies	Summary RR (95% CI)	*I*^2^,%	P*	P**
Geographic location					0.75
Europe	2	1.51 (1.20–1.91)	17.6	0.27	
Northern America	3	1.28 (0.12–14.07)	0.0	0.40	
Others	2	2.17 (1.05–4.52)	58.8	0.12	
Adjustment for confounders					
Maternal age					0.99
Yes	4	1.62 (1.20–2.18)	21.1	0.28	
No	3	1.64 (0.38–7.01)	29.0	0.25	
Ethnicity					0.48
Yes	2	1.89 (1.09–3.28)	0.0	0.55	
No	5	1.78 (0.94–3.39)	26.8	0.24	
Maternal education					0.32
Yes	3	1.46 (1.32–1.62)	0.0	0.54	
No	4	2.42 (0.78–7.49)	27.2	0.25	
Tobacco use					0.99
Yes	4	1.62 (1.20–2.18)	21.1	0.28	
No	3	1.64 (0.38–7.01)	29.0	0.25	
Alcohol drinking					0.16
Yes	2	2.50 (1.32–4.73)	2.3	0.31	
No	5	1.45 (1.31–1.61)	0.0	0.58	
Pregnancy BMI					0.54
Yes	2	1.51 (1.20–1.91)	17.6	0.27	
No	5	2.00 (0.94–4.28)	15.3	0.32	
Parity					0.32
Yes	3	1.46 (1.32–1.62)	0.0	0.54	
No	4	2.42 (0.78–7.49)	27.2	0.25	

BMI, body mass index; CI, confidence interval; RR, relative risk.

*P for heterogeneity within each subgroup.

**P for heterogeneity between subgroups with meta-regression analysis.

### SSRI Use in the General Pregnant Female Population

**Figure 4:**
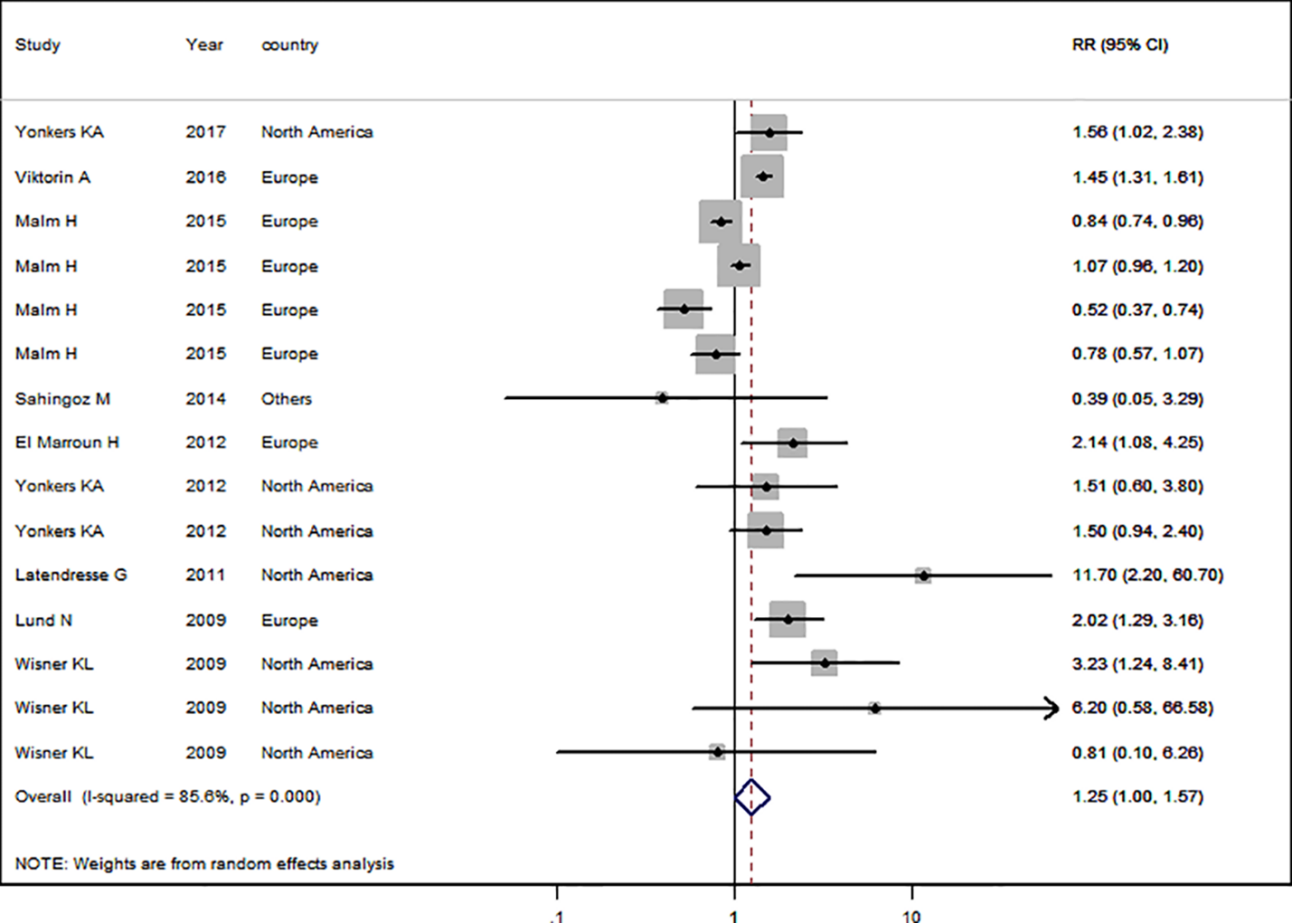
Meta-analysis of selective serotonin reuptake inhibitor use in pregnant women and the risk of preterm birth.

Nine studies (Lund et al., 2009; Wisner et al., 2009; Latendresse and Ruiz, 2011; El Marroun et al., 2012; Yonkers et al., 2012; Şahingöz et al., 2014; Malm et al., 2015; Viktorin et al., 2016; Yonkers et al., 2017) in the general pregnant female population were included for this analysis. The adjusted RR was 1.25 (95% CI 1.01−1.57, *I*^2^ = 85.6%, *P* < 0.001, **Figure 4**). The heterogeneity of the studies was high and no significantly increased risk of PTB was observed for SSRI use in the general pregnant female population. Subgroup analysis of SSRI use and risk of PTB is shown in **Table 4**. In four North American studies, the adjusted RR was 1.91 (1.27−2.86). Following adjustment for ethnicity, maternal education, alcohol consumption, and BMI during pregnancy, the adjusted RRs were statistically significant: 1.85 (1.33−2.57), 1.47 (1.33−1.62), 1.70 (1.19−2.44), and 1.63 (1.27−2.09), respectively. If parity was not adjusted, the RR was statistically significant [2.24 (1.03−4.84)]. There was no evidence of publication bias in any of the studies.

**Table 4:** Subgroup analysis of selective serotonin reuptake inhibitor use and risk of preterm birth in infants: results of meta-analyses.

	SSRI use				
	No. of studies	Summary RR (95% CI)	*I*^2^,%	P*	P**
Geographic location					0.22
Europe	4	1.07 (0.82–1.39)	92.30	< 0.001	
Northern America	4	1.91 (1.27–2.86)	33.6	0.17	
Others	1	0.39 (0.05–3.16)	–	–	
Adjustment for confounders					
Maternal age					0.34
Yes	7	1.22 (0.97–1.52)	86.4	< 0.001	
No	2	2.27 (0.08–63.58)	83.9	0.01	
Ethnicity					0.13
Yes	3	1.85 (1.33–2.57)	0.0	0.45	
No	6	1.11 (0.86–1.42)	89.4	< 0.001	
Maternal education					0.45
Yes	3	1.47 (1.33–1.62)	0.0	0.52	
No	6	1.14 (0.88–1.47)	79.7	< 0.001	
Tobacco use					0.10
Yes	6	1.16 (0.92–1.45)	89.0	< 0.001	
No	3	2.60 (0.86–7.84)	50.9	0.09	
Alcohol drinking					0.45
Yes	2	1.70 (1.19–2.44)	0.0	0.44	
No	7	1.18 (0.92–1.51)	86.9	< 0.001	
Pregnancy BMI					0.26
Yes	3	1.63 (1.27–2.09)	77.5	0.21	
No	6	1.09 (0.85–1.40)	0.4	< 0.001	
Parity					0.12
Yes	5	1.12 (0.88–1.43)	89.9	< 0.001	
No	4	2.24 (1.03–4.84)	52.0	0.06	

BMI, body mass index; CI, confidence interval; RR, relative risk; SSRI, selective serotonin reuptake inhibitor.

*P for heterogeneity within each subgroup.

**P for heterogeneity between subgroups with meta-regression analysis.

### SSRI Use in Pregnant Women With Depression

**Figure 5:**
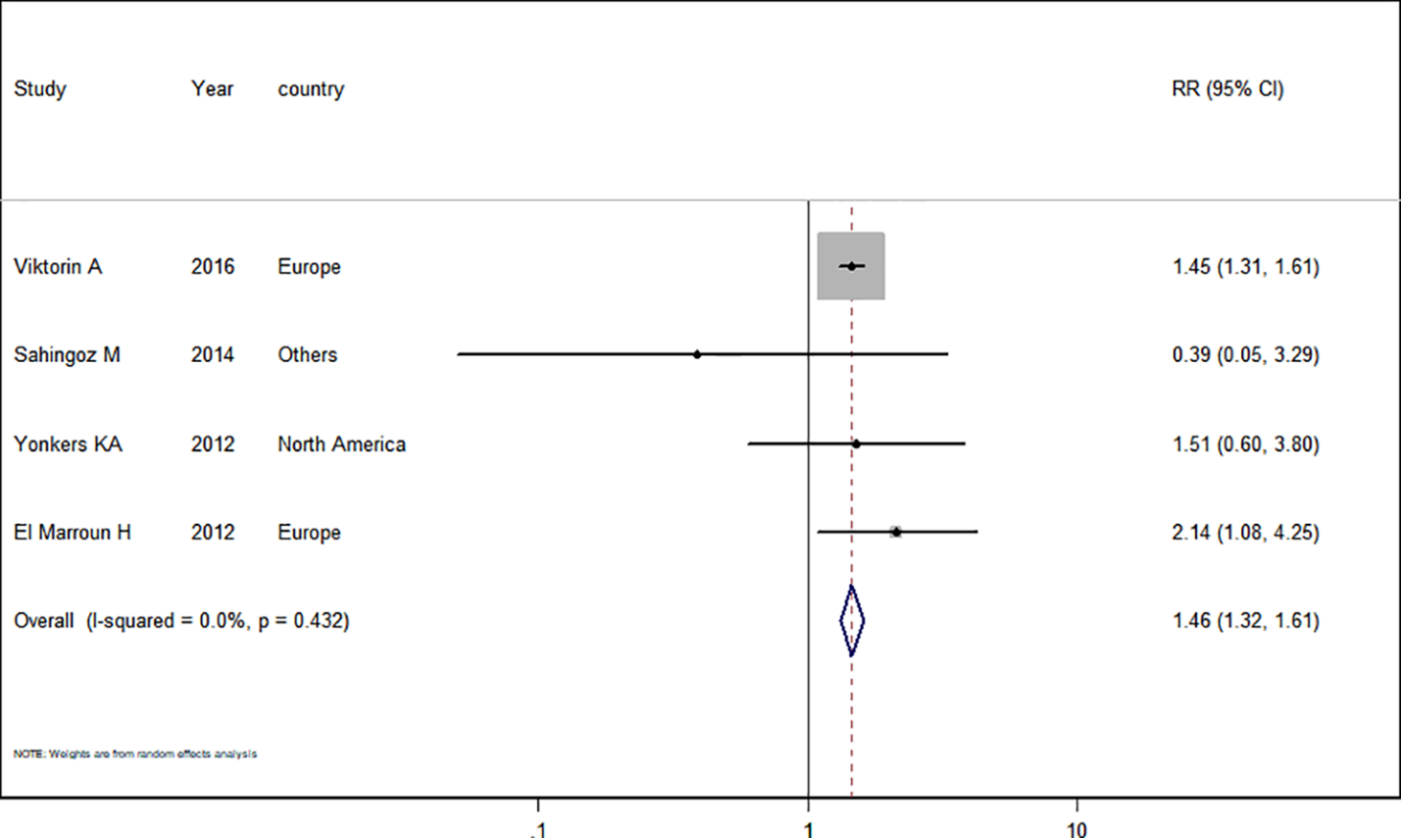
Meta-analysis of selective serotonin reuptake inhibitor use in pregnant women with depression and the risk of preterm birth.

Only four studies (El Marroun et al., 2012; Yonkers et al., 2012; Şahingöz et al., 2014; Viktorin et al., 2016) in the pregnant women with depression were included for this analysis. The adjusted RR was 1.46 (95% CI 1.32−1.61, *I*^2^ = 0.0%, *P* = 0.432, **Figure 5**). A significantly increased risk of PTB was observed with SSRI use in pregnant women with depression. There was no evidence of publication bias in any of the studies.

## Discussion

### Main Findings

To the best of our knowledge, this is the first meta-analysis of the impact of antidepressant use during pregnancy on PTB in depressed women. Our study focused not only on depressed pregnant women but also on the general pregnant female population, with 23 prospective cohort studies finally being selected. It was found that the risk of PTB was increased in depressed women treated with SSRIs during pregnancy. Similar results were observed in women with depression and in the general pregnant female population who used antidepressants during pregnancy. There was no significantly increased risk of PTB observed with SSRI use in the general pregnant female population.

The focus of the present study was to explore the association between maternal use of antidepressants in depressed women during pregnancy and risk of PTB. Subgroup analysis shows that even though the use of antidepressants in the general pregnant female population significantly increased the risk of PTB, the heterogeneity was high, which may be due to the differences in population sources. There was a statistically significant difference in the European cohort study but not in the North American cohort study, which may be one of the sources of heterogeneity. Another reason for the high heterogeneity may be that there were confounding factors that influenced the conclusion. For this reason, a meta-regression analysis was conducted; when maternal age, tobacco use, and parity were defined, there was a statistically significant difference in the meta-regression, indicating that these may be confounding factors.

To further explore whether the heterogeneity originated from different drug types, subgroup analysis of SSRI use in the general pregnant female population was conducted, and it was found that the heterogeneity was elevated. A possible reason could be differences in the sources of the population. Firstly, according to geographic location, studies were divided into European, North American, and others; the *P* values were 0.27, 0.40, and 0.12, respectively, and the meta-regression was not statistically significant. Subsequently, the data were adjusted for certain confounding factors such as maternal age, ethnicity, maternal education, tobacco use, and alcohol consumption; however, the results were not ideal. Finally, a subgroup analysis of SSRI use in pregnant women with depression was performed, and it was found that the heterogeneity was 0%; therefore, a possible source of the high heterogeneity was not stipulating depression in the study population. However, there have only been four studies to date, and the present study suggests that future studies may be inclined to stipulate depression in the study population and to consider the effects of SSRIs or other antidepressants on preterm PTB, which is of great clinical significance.

### Comparison With the Existing Literature

Currently, there exist four meta-analyses of the effects of antidepressant use on PTB (nearly 6 years) (Hsiang and Bridge, 2014; Huybrechts et al., 2014; Ross and Grigoriadis, 2014; Eke et al., 2016). The results of the present study among studies in general pregnant women were consistent with these published meta-analyses. However, these original cohort studies were both retrospective and prospective, which could have caused recall bias. Moreover, there was no stipulation of depression in the study population, with the exception of one study (Eke et al., 2016) including eight original studies, which focused on the effect of SSRIs on PTB in pregnant women with depression. Following adjustment for confounders, the incidence of PTB was significantly higher in pregnant women treated with SSRIs as compared with that in the controls [adjusted odds ratios (aOR) 1.24, 95% CI 1.09−1.41]. Our included studies were all prospective cohort studies with high quality evidence and high credibility. Huybrechts et al. (2014) included 41 original studies, and the pooled adjusted odds ratio was 1.53 (1.40−1.66) for antidepressant use at any time during pregnancy and 1.96 (1.62−2.38) for use during the third trimester; however, confounding factors were not adjusted for. In another study by Rose et al. (2014), 13 original studies providing 14 estimates of the association between antidepressant use and PTB resulted in a pooled OR of 1.55 (95% CI, 1.38−1.74; *P* < 0.001); after limiting the population to depressed pregnant women, the OR was 1.79 (0.77−4.14). Moreover, a study by Hsiang et al. (2014) included 28 original studies and the meta-analysis showed that antidepressant use in pregnancy was significantly associated with PTB (RR: 1.69, 95% CI: 1.52−1.88).

### Strengths and Limitations

The present study describes a meta-analysis of 23 prospective cohort studies investigating the risk of PTB in women taking antidepressants during pregnancy, with additional analyses associated with a diagnosis of depression. The main strength is that it is a large meta-analysis including a large number of prospective cohort studies. Furthermore, to-date, evidence regarding associations between the use of antidepressant in depressed women and risk of PTB is limited. To the best of our knowledge, the present study is the first meta-analysis to investigate the associations between maternal antidepressant use in depressed women and the risk of PTB.

Nevertheless, this study has several limitations, which should be considered when interpreting its findings. First, due to the limited number of included studies, the present study did not account for trimester exposure and either for duration of exposure in the analysis. Both factors may significantly modify the association between maternal use of antidepressant during pregnancy and PTB. The number of studies related to specific antidepressants are small for specific drugs (i.e., SSRIs) and did not allow for investigations into the dose-response. Second, heterogeneity was high among studies focusing on the associations between maternal antidepressant use in the general pregnant women and the risk of PTB. Our subgroup analysis suggested that the high heterogeneity might be attributed to differences in study locations and diagnosing of depression.

## Conclusions

The results of this meta-analysis highlights the association between maternal use of antidepressant during pregnancy and the risk of PTB. Health care providers and pregnant women must weigh the risk-benefit potential of these drugs when making decisions about whether to treat with antidepressant during pregnancy.

## Author Contributions

Y-HZ and QC designed and conducted the study. X-YM and X-XR collected, managed, and analyzed the data. QC, Q-JW, HS, and Y-HZ prepared, reviewed, and approved the manuscript. Y-HZ had full access to all the data in the study and takes responsibility for the integrity of the data and the accuracy of the data analysis.

## Funding

This work was supported by National Key R&D Program of China (No. 2017YFC0907403 to Y-HZ); Liaoning Revitalization Talents Program (No. XLYC1802095 to Y-HZ); Key R&D Program of Liaoning Province (No. 2019JH8/10300005 to Y-HZ); the Science and Technology Project of Liaoning Province (No. 2019JH6/10400002 to Y-HZ); Young Talents of Education Ministry of Liaoning Province (No. QN2019011 to HS). The other authors declare no conflicts of interest in relation to this study.

## Conflict of Interest

The authors declare that the research was conducted in the absence of any commercial or financial relationships that could be construed as a potential conflict of interest.

## References

[B1] Şahingöz M. Yuksel G. Karsidag C. Uguz F. Sonmez E. O. Annagur B. B. (2014). Birth Weight and Preterm Birth in Babies of Pregnant Women With Major Depression in Relation to Treatment With Antidepressants. J. Clin. Psychopharmacol. 34 (2), 226–229. 10.1097/JCP.0000000000000077 24525643

[B2] Begg C. B. Mazumdar M. (1994). Operating characteristics of a rank correlation test for publication bias. Biometrics 50 (4), 1088–1101. 10.2307/2533446 7786990

[B3] Blencowe H. Cousens S. Oestergaard M. Z. Chou D. Moller A. B. Narwal R. (2012). National, regional, and worldwide estimates of preterm birth rates in the year 2010 with time trends since 1990 for selected countries: a systematic analysis and implications. Lancet 379 (9832), 2162–2172. 10.1016/S0140-6736(12)60820-4 22682464

[B4] Calderon-Margalit R. Qiu C. Ornoy A. Siscovick D. S. Williams M. A. (2009). Risk of preterm delivery and other adverse perinatal outcomes in relation to maternal use of psychotropic medications during pregnancy. Am. J. Psychiatry 201 (6), 557–566. 10.1016/j.ajog.2009.06.061 PMC2881461 19691950

[B5] Chang H. H. Larson J. Blencowe H. Spong C. Y. Howson C. P. Cairns-Smith S. (2013). Preventing preterm births: analysis of trends and potential reductions with interventions in 39 countries with very high human development index. Lancet 381 (9862), 223–234. 10.1016/S0140-6736(12)61856-X 23158883 PMC3572865

[B6] DerSimonian R. Laird N. (1986). Meta-analysis in clinical trials. Control Clin. Trials. 7 (3), 177–188. 10.1016/0197-2456(86)90046-2 3802833

[B7] Djulus J. Koren G. Einarson T. R. Wilton L. Shakir S. Diav-Citrin O. (2006). Exposure to mirtazapine during pregnancy: a prospective, comparative study of birth outcomes. J. Clin. Psychiatry 67 (8), 1280–1284. 10.4088/JCP.v67n0817 16965209

[B8] Einarson A. Choi J. Einarson T. R. Koren G. (2010). Adverse effects of antidepressant use in pregnancy: an evaluation of fetal growth and preterm birth. Depress Anxiety 27 (1), 35–38. 10.1002/da.20598 19691030

[B9] Einarson A. Choi J. Koren G. Einarson T. (2011). Outcomes of infants exposed to multiple antidepressants during pregnancy: Results of a cohort study. J. Popul. Ther. Clin. Pharmacol. 18 (2), e390–e396. 22071601

[B10] Eke A. C. Saccone G. Berghella V. (2016). Selective Serotonin Reuptake Inhibitor (SSRI) Use During Pregnancy and Risk of Preterm Birth: A Systematic Review and Meta-Analysis. BJOG 123 (12), 1900–1907. 10.1111/1471-0528.14144 27239775 PMC9987176

[B11] El Marroun H. Jaddoe V. W. Hudziak J. J. Roza S. J. Steegers E. A. Hofman A. (2012). Maternal Use of Selective Serotonin Reuptake Inhibitors, Fetal Growth, and Risk of Adverse Birth Outcomes. Arch. Gen. Psychiatry 69 (7), 706–714. 10.1001/archgenpsychiatry.2011.2333 22393202

[B12] El Marroun H. White T. Verhulst F. C. Tiemeier H. (2014). Maternal use of antidepressant or anxiolytic medication during pregnancy and childhood neurodevelopmental outcomes: a systematic review. Eur. Child Adolesc. Psychiatry 23 (10), 973–992. 10.1007/s00787-014-0558-3 24863148

[B13] Gao S. Y. Wu Q. J. Sun C. Zhang T. N. Shen Z. Q. Liu C. X. (2018). Selective serotonin reuptake inhibitor use during early pregnancy and congenital malformations: a systematic review and meta-analysis of cohort studies of more than 9 million births. BMC Med. 12;16 (1), 205. 10.1186/s12916-018-1193-5 PMC6231277 30415641

[B14] Higgins J. P. T. Green S. (2011). Cochrane Handbook for Systematic Reviews of Interventions, version 5.1.0. The Cochrane Collaboration Available at: https://training.cochrane.org/handbook#how-to-access (Accessed May 13, 2020).

[B15] Hsiang H. Bridge J. A. (2014). A meta-analysis of the relationship between antidepressant use in pregnancy and the risk of preterm birth and low birth weight: letter response. Gen. Hosp. Psychiatry 36 (3), 358–359. 10.1016/j.genhosppsych.2014.01.005 24556258

[B16] Huybrechts K. F. Sanghani R. S. Avorn J. Urato A. C. (2014). Preterm Birth and Antidepressant Medication Use during Pregnancy: A Systematic Review and Meta-Analysis. PloS One 9 (3), e92778. 10.1371/journal.pone.0092778 24671232 PMC3966829

[B17] Källén B. (2004). Neonate Characteristics After Maternal Use of Antidepressants in Late Pregnancy. Arch. Pediatr. Adolesc. Med. 158 (4), 312–316. 10.1001/archpedi.158.4.312 15066868

[B18] Klieger-Grossmann C. Weitzner B. Panchaud A. Pistelli A. Einarson T. Koren G. (2012). Pregnancy Outcomes Following Use of Escitalopram: A Prospective Comparative Cohort Study. J. Clin. Pharmacol. 52 (5), 766–770. 10.1177/0091270011405524 22075232

[B19] Laine M. K. Masalin S. Rönö K. Kautiainen H. Gissler M. Pennanen P. (2019). Risk of preterm birth in primiparous women with exposure to antidepressant medication before pregnancy and/or during pregnancy-impact of body mass index. Ann. Med. 51 (1), 51–57. 10.1080/07853890.2018.1534265 30299166 PMC7857451

[B20] Latendresse G. Ruiz R. J. (2011). Maternal corticotropin-releasing hormone and the use of selective serotonin reuptake inhibitors independently predict the occurrence of preterm birth. J. Midwifery Womens Health 56 (2), 118–126. 10.1111/j.1542-2011.2010.00023.x 21429075 PMC3077095

[B21] Lee A. M. Lam S. K. Sze Mun Lau S. M. Chong C. S. Chui H. W. Fong D. Y. (2007). Prevalence, course, and risk factors for antenatal anxiety and depression. Obstet. Gynecol. 110 (5), 1102–1112. 10.1097/01.AOG.0000287065.59491.70 17978126

[B22] Lewis A. J. Galbally M. Opie G. Buist A. (2010). Neonatal growth outcomes at birth and one month postpartum following in utero exposure to antidepressant medication. Aust. N. Z. J. Psychiatry 44 (5), 482–487. 10.3109/00048670903559593 20397792

[B23] Lund N. Pedersen L. H. Henriksen T. B. (2009). Selective Serotonin Reuptake Inhibitor Exposure In Utero and Pregnancy Outcomes. Arch. Pediatr. Adolesc. Med. 163 (10), 949–954. 10.1001/archpediatrics.2009.164 19805715

[B24] Malm H. Sourander A. Gissler M. Gyllenberg D. Hinkka-Yli-Salomäki S. McKeague I. W. (2015). Pregnancy Complications Following Prenatal Exposure to SSRIs or Maternal Psychiatric Disorders: Results From Population-Based National Register Data. Am. J. Psychiatry 172 (12), 1224–1232. 10.1176/appi.ajp.2015.14121575 26238606

[B25] Mangham L. J. Petrou S. Doyle L. W. Draper E. S. Marlow N. (2009). The Cost of Preterm Birth Throughout Childhood in England and Wales. Pediatrics 123 (2), e312–e327. 10.1542/peds.2008-1827 19171583

[B26] Marlow N. Wolke D. Bracewell M. A. Samara M. EPICure Study Group (2000). Neurologic and Developmental Disability at Six Years of Age after Extremely Preterm Birth. N. Engl. J. Med. 6 (1), 4–5. 10.1056/NEJMoa041367 15635108

[B27] McDonald S. D. Han Z. Mulla S. Beyene J. (2010). Knowledge Synthesis Group. Overweight and obesity in mothers and risk of preterm birth and low birth weight infants: systematic review and meta-analyses. BMJ 341, c3428. 10.1136/bmj.c3428 20647282 PMC2907482

[B28] Nordeng H. van Gelder M. M. Spigset O. Koren G. Einarson A. Eberhard-Gran M. (2012). Pregnancy Outcome After Exposure to Antidepressants and the Role of Maternal Depression. J. Clin. Psychopharmacol. 32 (2), 186–194. 10.1097/JCP.0b013e3182490eaf 22367660

[B29] Odutayo A. Wong C. X. Hsiao A. J. Hopewell S. Altman D. G. Emdin C. A. (2016). Atrial fibrillation and risks of cardiovascular disease, renal disease, and death:systematic review and meta-analysis. BMJ 354, i4482. 10.1136/bmj.i4482 27599725

[B30] Purisch S. E. Gyamfi-Bannerman C. (2017). Epidemiology of preterm birth. Semin. Perinatol. 41 (7), 387–391. 10.1053/j.semperi.2017.07.009 28865982

[B31] Richardson J. L. Martin F. Dunstan H. Greenall A. Stephens S. Yates L. M. (2019). Pregnancy outcomes following maternal venlafaxine use: A prospective observational comparative cohort study. Reprod. Toxicol. 84, 108–113. 10.1016/j.reprotox.2019.01.003 30639403

[B32] Ross L. E. Grigoriadis S. (2014). Selected Pregnancy and Delivery Outcomes After Exposure to Antidepressant Medication. JAMA Psychiatry 71 (6), 716. 10.1001/jamapsychiatry.2014.59 24898812

[B33] Sadowski A. Todorow M. Yazdani Brojeni P. Koren G. Nulman I. (2013). Pregnancy outcomes following maternal exposure to second-generation antipsychotics given with other psychotropic drugs: a cohort study. BMJ Open 3 (7), e003062. 10.1136/bmjopen-2013-003062 PMC3710985 23852139

[B34] Sivojelezova A. Shuhaiber S. Sarkissian L. Einarson A. Koren G. (2005). Citalopram use in pregnancy: Prospective comparative evaluation of pregnancy and fetal outcome. Am. J. Obstet. Gynecol. 193 (6), 2004–2009. 10.1016/j.ajog.2005.05.012 16325604

[B35] Staneva A. Bogossian F. Pritchard M. Wittkowski A. (2015). The effects of maternal depression, anxiety, and perceived stress during pregnancy on preterm birth: A systematic review. Women Birth. 28 (3), 179–193. 10.1016/j.wombi.2015.02.003 25765470

[B36] Suri R. Altshuler L. Hellemann G. Burt V. K. Aquino A. Mintz J. (2007). Effects of Antenatal Depression and Antidepressant Treatment on Gestational Age at Birth and Risk of Preterm Birth. Am. J. Psychiatry 164 (8), 1206–1213. 10.1176/appi.ajp.2007.06071172 17671283

[B37] Viktorin A. Lichtenstein P. Lundholm C. Almqvist C. D'Onofrio B. M. Larsson H. (2016). Selective serotonin re-uptake inhibitor use during pregnancy: association with offspring birth size and gestational age. Int. J. Epidemiol. 45 (1), 170–177. 10.1093/ije/dyv351 26748846

[B38] Wells G. A. Shea B. J. O'Connell D. Peterson J. Welch V. Losos M. (2011). Newcastle–Ottawa scale (NOS) for assessing the quality of nonrandomized studies in meta-analysis. Available at: www.ohri.ca/programs/clinical_epidemiology/oxford.asp (Accessed May 13, 2020).

[B39] Winterfeld U. Klinger G. Panchaud A. Stephens S. Arnon J. Malm H. (2015). Pregnancy Outcome Following Maternal Exposure to Mirtazapine: A Multicenter, Prospective Study. J. Clin. Psychopharmacol. 35 (3), 250–259. 10.1097/JCP.0000000000000309 25830592

[B40] Wisner K. L. Sit D. K. Hanusa B. H. Bogen D. L. Hunker D. F. Perel J. M. (2009). Major depression and antidepressant treatment: impact on pregnancy and neonatal outcomes. Am. J. Psychiatry 166 (5), 557–566. 10.1176/appi.ajp.2008.08081170 19289451 PMC4426499

[B41] Yonkers K. A. Norwitz E. R. Smith M. V. Lockwood C. J. Gotman N. Luchansky E. (2012). Depression and Serotonin Reuptake Inhibitor Treatment as Risk Factors for Preterm Birth. Epidemiology 23 (5), 677–685. 10.1097/EDE.0b013e31825838e9 22627901 PMC3415566

[B42] Yonkers K. A. Gilstad-Hayden K. Forray A. Lipkind H. S. (2017). Association of Panic Disorder, Generalized Anxiety Disorder, and Benzodiazepine Treatment During Pregnancy With Risk of Adverse Birth Outcomes. JAMA Psychiatry 74 (11), 1145–1152. 10.1001/jamapsychiatry.2017.2733 28903165 PMC5710298

