# Supplementary material for: Antidepressant Use in Depressed Women During Pregnancy and the Risk of Preterm Birth: A Systematic Review and Meta-Analysis of 23 Cohort Studies
Source: Front Pharmacol. 2020 May 19;11:659. doi: 10.3389/fphar.2020.00659 (PMC7250148; doi:10.3389/fphar.2020.00659)
Supplement: Supplementary file 1 [file DataSheet_1.docx]

**Supplementary file 1.** Search queries used in the three databases.

**(1) PubMed**

(((((((Agents, Antidepressive) OR (Antidepressant Drugs) OR (Drugs, Antidepressant) OR (Antidepressants) OR (Thymoanaleptics) OR (Thymoleptics))) OR ((Tricyclic Antidepressive Agents) OR (Agents, Tricyclic Antidepressive) OR (Antidepressant Drugs, Tricyclic ) OR (Drugs, Tricyclic Antidepressant) OR (Tricyclic Antidepressant Drugs) OR (Antidepressants, Tricyclic) OR (Tricyclic Antidepressants))) OR ((5-Hydroxytryptamine Uptake Inhibitors) OR (5 Hydroxytryptamine Uptake Inhibitors) OR (Inhibitors, 5-HT Uptake) OR (Inhibitors, 5 HT Uptake) OR (Inhibitors, 5-Hydroxytryptamine Uptake) OR (Inhibitors, 5 Hydroxytryptamine Uptake) OR (Inhibitors, Serotonin Reuptake) OR (Reuptake Inhibitors, Serotonin) OR (Serotonin Reuptake Inhibitors) OR (Uptake Inhibitors, 5-HT) OR (Uptake Inhibitors, 5 HT) OR (Uptake Inhibitors, 5-Hydroxytryptamine) OR (Uptake Inhibitors, 5 Hydroxytryptamine) OR (Uptake Inhibitors, Serotonin) OR (5-HT Uptake Inhibitors) OR (5 HT Uptake Inhibitors) OR (Inhibitors, Serotonin Uptake) OR (Selective Serotonin Reuptake Inhibitors))) OR ((SSRIs and NRIs) OR (NRIs and SSRIs) OR (Serotonin and Noradrenaline Uptake Inhibitors) OR (SNRIs) OR (Serotonin and Norepinephrine Reuptake Inhibitors) OR (Serotonin and Norepinephrine Uptake Inhibitors)))) OR ("Noradrenergic and Specific Serotonergic Antidepressants" OR "NaSSAs" OR "Norepinephrine dopamine reuptake inhibitor" OR "NDRI" OR "Selective Noradrenalin Reuptake Inhibitor" OR "NARIs" OR "Serotonin Antagonist Reuptake Inhibitor" OR "SARIs")

AND

((Birth, Premature) OR (Births, Premature) OR (Premature Births) OR (Preterm Birth) OR (Birth, Preterm) OR (Births, Preterm) OR (Preterm Births) OR (Pregnancy Outcomes) OR (Outcome, Pregnancy) OR (Outcomes, Pregnancy))

**(2) PsycINFO**

(TX antidepressant drugs OR TX Tricyclic Antidepressant Drugs OR TX serotonin uptake inhibitors OR TX Serotonin Reuptake Inhibitors OR TX Serotonin Norepinephrine Reuptake Inhibitors OR TX (Noradrenergic and Specific Serotonergic Antidepressants) OR TX NaSSAs OR TX Norepinephrine dopamine reuptake inhibitor OR TX NDRI OR TX Serotonin Antagonist Reuptake Inhibitor OR TX SARI)

AND

(TX Premature Birth OR TX pregnancy outcomes)

**(3) EMBASE**

('antidepressant agent' OR ‘anti depressant agent’ OR ‘antidepressant’ OR ‘antidepressant drug’ OR ‘antidepressants’ OR ‘antidepressants, miscellaneous’ OR ‘antidepression drug’ OR ‘antidepressive agent’ OR ‘antidepressive agents’ OR ‘antidepressive agents, second generation’ OR ‘antidepressive agents, second-generation’ OR ‘antidepressive drug’ OR ‘neurothymoleptic agent’ OR ‘psychoenergizer’ OR ‘thymoleptic’ OR ‘thymoleptic agent’ OR ‘thymoleptic drug’ OR ‘thymolytic agent’ OR ‘tricyclic antidepressant agent’ OR ‘antidepressant, tricyclic’ OR ‘antidepressants, tricyclic’ OR ‘antidepressive agents, tricyclic’ OR ‘tricyclic antidepressant’ OR ‘tricyclic antidepressants’ OR ‘tricyclic antidepressive agent’ OR ‘serotonin uptake inhibitor’ OR ‘antidepressants, serotonin specific reuptake inhibitors’ OR ‘selective serotonin reuptake inhibitor’ OR ‘serotonin reuptake inhibitor’ OR ‘serotonin specific reuptake inhibitor’ OR ‘serotonin specific reuptake inhibitors’ OR ‘serotonin uptake inhibitors’ OR ‘SSRI’ OR ‘SSRI antidepressant’ OR ‘serotonin noradrenalin reuptake inhibitor’ OR ‘dual monoamine reuptake inhibitor’ OR ‘dual monoamine uptake inhibitor’ OR ‘dual reuptake inhibitor’ OR ‘dual uptake inhibitor’ OR ‘noradrenalin serotonin reuptake inhibitor’ OR ‘noradrenalin serotonin uptake inhibitor’ OR ‘norepinephrine serotonin reuptake inhibitor’ OR ‘norepinephrine serotonin uptake inhibitor’ OR ‘selective serotonin noradrenalin reuptake inhibitor’ OR ‘serotonin and noradrenaline reuptake inhibitor’ OR ‘serotonin and noradrenaline reuptake inhibitors’ OR ‘serotonin and noradrenaline uptake inhibitor’ OR ‘serotonin and norepinephrine reuptake inhibitor’ OR ‘serotonin and norepinephrine uptake inhibitor’ OR ‘serotonin noradrenalin uptake inhibitor’ OR ‘serotonin norepinephrine reuptake inhibitor’ OR ‘serotonin norepinephrine uptake inhibitor’ OR ‘SNRI’ OR ‘SNRIs’ OR ‘SSNRI’ OR ‘noradrenalin uptake inhibitor’ OR ‘noradrenalin reuptake inhibitor’ OR ‘noradrenaline reuptake inhibitor’ OR ‘noradrenaline uptake inhibitor’ OR ‘norepinephrine reuptake inhibitor’ OR ‘norepinephrine uptake inhibitor’ OR ‘selective noradrenalin reuptake inhibitor’ OR ‘selective noradrenalin uptake inhibitor’ OR ‘selective norepinephrine reuptake inhibitor’ OR ‘selective norepinephrine uptake inhibitor’ OR 'noradrenergic and specific serotonergic antidepressants' OR 'nassas' OR 'norepinephrine dopamine reuptake inhibitor' OR 'ndri' OR 'serotonin antagonist reuptake inhibitor' OR 'saris')

AND

(‘premature labor’ OR ‘labor, premature’ OR ‘labour, premature’ OR ‘obstetric labor, premature’ OR ‘obstetric labour, premature’ OR ‘premature delivery’ OR ‘premature labour’ OR ‘preterm birth’ OR ‘preterm delivery’ OR ‘preterm labor’ OR ‘preterm labour’ OR ‘pregnancy outcome’ OR ‘birth outcome’ OR ‘obstetric outcome’)
