# Supplementary material for: Antidepressant Use in Depressed Women During Pregnancy and the Risk of Preterm Birth: A Systematic Review and Meta-Analysis of 23 Cohort Studies
Source: Front Pharmacol. 2020 May 19;11:659. doi: 10.3389/fphar.2020.00659 (PMC7250148; doi:10.3389/fphar.2020.00659)
Supplement: Supplementary file 2 [file DataSheet_2.doc]

| **Supplementary file2. Risk of bias of included studies as assessed with the Newcastle-Ottawa Scale** | | | | | |
| --- | --- | --- | --- | --- | --- |
| **Author, year** | **Selection (4)** | **Comparability (2)** | **Outcome (3)** | **Total (9)** | **Comments** |
| Richardson et al., 2019 | 3 | 1 | 3 | 7 | Outcomes ascertained through telephone interview |
| Laine et al., 2019 | 4 | 1 | 3 | 8 |  |
| Yonkers et al., 2017 | 4 | 1 | 3 | 8 | Outcomes ascertained through medical records |
| Viktorin et al., 2016 | 4 | 2 | 3 | 9 |  |
| Winterfeld et al., 2015 | 3 | 1 | 3 | 7 | Outcomes ascertained through telephone interview |
| Malm et al., 2015 | 4 | 2 | 3 | 9 | Outcomes ascertained through record linkage |
| Şahingöz et al., 2014 | 3 | 1 | 3 | 7 | Outcomes ascertained through medical records |
| Sadowski et al., 2013 | 3 | 1 | 3 | 7 | Outcomes ascertained through telephone interview |
| Nordeng et al., 2012 | 4 | 2 | 3 | 9 |  |
| Yonkers et al., 2012 | 4 | 2 | 3 | 9 | Outcomes ascertained through medical records |
| El Marroun et al., 2012 | 3 | 2 | 3 | 8 | Outcomes ascertained through asking |
| Klieger-Grossmann et al., 2012 | 3 | 1 | 3 | 7 | Outcomes ascertained through telephone interview |
| Einarson et al., 2011 | 3 | 1 | 3 | 7 | Outcomes ascertained through telephone interview |
| Latendresse et al., 2011 | 3 | 0 | 3 | 6 | Outcomes ascertained through record linkage |
| Einarson et al., 2010 | 3 | 1 | 3 | 7 | Outcomes ascertained through telephone interview |
| Lewis et al., 2010 | 4 | 1 | 3 | 8 |  |
| Lund et al., 2009 | 4 | 2 | 3 | 9 |  |
| **Author, year** | **Selection (4)** | **Comparability (2)** | **Outcome (3)** | **Total (9)** | **Comments** |
| Wisner et al., 2009 | 4 | 2 | 3 | 9 | Outcomes ascertained through medical records |
| Calderon-Margalit et al., 2009 | 4 | 2 | 3 | 9 |  |
| Suri et al., 2007 | 4 | 1 | 3 | 8 | Outcomes ascertained through medical records |
| Djulus et al., 2006 | 3 | 1 | 3 | 7 | Outcomes ascertained through telephone interview |
| Sivojelezova et al., 2005 | 3 | 1 | 3 | 7 | Outcomes ascertained through telephone interview |
| Källén et al., 2004 | 4 | 0 | 3 | 7 | Did not control for any factor |
